# Cleaning Products Commonly Used in Oklahoma Family Child Care Homes: Implications for Respiratory Risk and Children’s Health

**DOI:** 10.3390/ijerph19074299

**Published:** 2022-04-03

**Authors:** Cassandra D. Querdibitty, Marianna S. Wetherill, Susan B. Sisson, Bethany Williams, Kan Aithinne, Haeyn Seo, Nancy R. Inhofe, Janis Campbell, Megan Slawinski, Alicia L. Salvatore

**Affiliations:** 1 Department of Health Promotion Sciences, Hudson College of Public Health, University of Oklahoma Health Sciences Center, 801 N.E. 13th Street, Oklahoma City, OK 73104, USA; cassandra-querdibitty@ouhsc.edu (C.D.Q.); marianna-wetherill@ouhsc.edu (M.S.W.); haeynseo@gmail.com (H.S.); mslawinski@tactilemedical.com (M.S.); 2 Department of Nutritional Sciences, College of Allied Health, University of Oklahoma Health Sciences Center, 1200 N. Stonewall Ave., Oklahoma City, OK 73114, USA; susan-sisson@ouhsc.edu (S.B.S.); bethany.williams1@wsu.edu (B.W.); 3 Department of Nutrition and Exercise Physiology, Elson S. Floyd College of Medicine, Washington State University Health Sciences Spokane, 412 E. Spokane Falls Blvd., Spokane, WA 99202, USA; 4 Department of Occupational and Environmental Health, Hudson College of Public Health, University of Oklahoma Health Sciences Center, 801 N.E. 13th Street, Oklahoma City, OK 73104, USA; k.aithinne@gmail.com; 5 Department of Pediatrics, College of Medicine, University of Oklahoma Health Sciences Center-Tulsa, 4444 E. 41st Street, Tulsa, OK 74135, USA; nancy-inhofe@ouhsc.edu; 6 Department of Biostatistics and Epidemiology, Hudson College of Public Health, University of Oklahoma Health Sciences Center, 801 N.E. 13th Street, Oklahoma City, OK 73104, USA; janis-campbell@ouhsc.edu; 7 Institute for Research on Equity and Community Health (iREACH), Christiana Care, Avenue North, 4000 Nexus Drive, CEI-300, Wilmington, DE 19803, USA; 8 Department of Human Development and Family Sciences, University of Delaware, 111 Alison Hall West, Newark, DE 19716, USA

**Keywords:** Oklahoma, child care, early care and education, children’s environmental health, chemicals, asthma

## Abstract

Little is known about the cleaning products used by early care and education programs that contribute to childhood asthma, particularly in Oklahoma where rates of uncontrolled asthma are higher than national rates (60.0% vs. 50.3%, respectively). We conducted a cross-sectional study of cleaning products used by Oklahoma-licensed family child care homes (FCCHs) (*n* = 50) to characterize and identify potential respiratory-health risks associated with chemical contents. Overall, 386 chemicals were abstracted from the 132 reported products. Of these, 100 unique chemicals were identified. Four percent (4.2%) of providers used a product with a sensitizer that may cause allergy or asthma symptoms if inhaled and 35.4% used a product with an irritant that may cause irritation to the respiratory tract. Most (62.5%) reported using a product with a chemical that had a C=C double bond in its molecular structure that may make it highly reactive with other substances in the air and produce secondary air pollutants and 83.3% reported using a sodium hypochlorite containing product. Twenty-three percent reported products that contain carcinogens. Policy, educational, and technical assistance interventions are needed to promote the use of safer products and reduce respiratory and other health risks posed by chemicals in Oklahoma FCCHs.

## 1. Introduction

Early care and education (ECE) programs, also known as child care, play a critical role in shaping children’s health and development. Approximately 61% of children under the age of five in the United States (US) receive regular care in ECE programs [1], with some spending up to 50 h per week in these environments [2,3]. State licensing regulations require ECE programs to prevent and control disease transmission, primarily through cleaning, sanitizing, and disinfecting [4]; however, products used for these purposes may routinely expose children to environmental toxicants [3,5,6,7]. Although effectively disinfecting and sanitizing is important for ensuring hygienic ECE environments, products such as bleach may pose a risk to children’s respiratory and overall health [8]. Children are especially vulnerable to environmental toxicants due to their (i) higher respiratory rates that cause them to breathe a larger volume of air per unit body weight [9]; (ii) increased physical exertion that causes them to breathe in more through their mouths, resulting in a greater volume of harmful chemicals to be inhaled [10]; and (iii) developing organ systems, with their lungs remaining undeveloped until adolescence [11,12]. Children’s small stature also puts their breathing zone closer to the floor where toxic gases tend to collect, resulting in higher inhalation of toxicants. Frequent use of cleaning products in ECE environments may increase children’s exposure to a variety of harmful environmental toxicants including volatile organic compounds (VOCs), particulate matter (PM), and nitrogen dioxide, which have been associated with decreased lung function, inflammation, airway obstruction, increased allergen sensitization, and the exacerbation of asthma symptoms [13,14,15,16,17,18,19].

According to the US Environmental Protection Agency (EPA), cleaners are intended to be used for removing dirt and organic matter from surfaces using detergents; sanitizers are intended to be used for killing or inactivating bacteria using chemicals; and disinfectants are intended to be used for killing or inactivating viruses and bacteria using chemicals [20]. Sanitizing and disinfecting products are regulated by the US EPA and registered as antimicrobial pesticides. Cleaning products are only required to be registered with the US EPA if they sanitize or disinfect (i.e., contain antimicrobial pesticides); therefore most are not registered. Cleaning products, particularly sprays, contain a complex mixture of hazardous chemicals including VOCs [21]. Aerosols or pump sprays also emit chemicals as gases and aerosols that may interact with other compounds in the indoor air, creating secondary toxicants such as PM [22,23,24]. Most state licensing regulations require ECE programs to use bleach or an EPA-registered disinfectant [25]. Bleach, which is the most commonly used product in ECE settings [5,26], contains chlorine-releasing antimicrobial agents (i.e., sodium hypochlorite). These agents can interact with toxicants in other cleaning products such as ammonia and acids to create nitrogenated compounds (e.g., chloramines and others) that may cause respiratory irritation [27,28]. Formaldehyde, which is another disinfecting byproduct of bleach [29], is a known irritant of the eyes, nose, throat, and lungs, and can act as a trigger of asthma symptoms; prolonged exposures can cause cancer [30]. Bleach also emits small amounts of chloroform, another known carcinogen, and at high temperatures, can emit up to twice as much chloroform [29].

ECE licensing regulations require many surfaces to be sanitized or disinfected daily and others, such as changing tables, to be disinfected after each use, frequently exposing children to toxicants. To illustrate, healthy infants have eight or more diaper changes a day, so changing tables at ECE programs caring for multiple infants may be used numerous times an hour [31] and disinfected after each use, usually with bleach or bleach-containing sprays, while children are present. VOCs are known to stay in the air for up to 20 min after a cleaning task is completed [32]. ECEs that maintain a rigid cleaning schedule that allows bleach the appropriate dwell time of 10 min before each diaper change could potentially expose children to lingering VOCs in the air for at least 30 min per diaper change [29]. Although limited, environmental sampling studies have documented the presence of environmental toxicants in ECE settings [3,5,26,33]. All four US studies [3,5,26,33], documented the presence of VOCs such as chloroform, benzene, and ethylbenzene, some above health-based limits. Studies conducted outside of the US have also documented the presence of environmental toxicants in ECE settings [34,35,36,37]. Notably, a study of nurseries in Portugal found high levels of formaldehyde and VOCs with levels peaking at the beginning of the morning, during or immediately after lunch, and in the afternoon [34], suggesting that toxicants originated from indoor sources such as cleaning products [34].

Understanding the types of cleaning products used in ECEs and the potential health impacts of the chemicals found in these products is critical to developing interventions to reduce children’s risk and create healthier ECE environments. Yet, little is known about the types of cleaning products that are used by ECE providers. Most studies conducted to date have measured air quality or chemical residues or assessed ECE providers’ general cleaning practices [3,5,26,33]. Most of this research has been conducted in child care centers (i.e., community or center-based child care and Head Start), which are usually located in large facilities with multiple classes, large class sizes, and multiple staff members [4]. Little research has been conducted in family child care homes (FCCHs) or home-based child care, which are located in residences, usually have a single provider, and care for no more than 12 children at a time [38]. To the best of our knowledge, only one study, which was conducted outside of the US, examined cleaning products used in ECEs and their potential health impacts [6]. This nationwide survey of more than 300 nurseries, kindergartens, and elementary schools across France found that 584 different cleaning products were used; safety data sheets (SDSs) were available for 218 products. Of the 152 chemicals abstracted from the products’ SDSs, more than half of the chemicals were respiratory irritants and seven percent were reactive chemicals containing a C=C double bonds that can react easily with environmental ozone to produce secondary air pollutants that may pose respiratory and other health risks, such as aldehydes and PM [6,22,23,39]. In this study, conducted among Oklahoma City metropolitan area licensed FCCH providers (*n* = 50), we characterized cleaning products used and identified potential health risks associated with their chemical contents, while in Oklahoma, licensed FCCHs and centers are bound to the same requirements, prior research conducted by our research team in Oklahoma indicates that FCCHs’ reported cleaning and other related practices differ from those reported by centers [7].

## 2. Materials and Methods

### 2.1. Study Design

This study used baseline data collected in Happy Healthy Homes, a randomized, matched-attention, controlled intervention trial aimed at FCCH providers (*n* = 50); a detailed study protocol has been previously published [40] and the trial is registered with Clinicaltrials.gov (NCT03560050). FCCH providers were: (i) licensed FCCHs serving at least one 2-to-5-year-old child; (ii) caring for low-income children and participating in the Child and Adult Care Food Program; (iii) located within the Oklahoma City metropolitan area (approximately a 60-mile radius); and (iv) planning to remain in business for at least 12 months. Providers were recruited through sponsor organizations and direct phone calls. A total of 370 eligible FCCH providers were originally contacted, of which 74 providers were screened and consented; 50 providers completed baseline data collection. Trained research staff visited participating FCCHs to collect baseline data, including provider surveys, environmental observations and product label abstraction, from October 2017 through October 2018. The study was approved by the University of Oklahoma Health Sciences Center Institutional Review Board.

### 2.2. Measures

#### 2.2.1. Participant Characteristics

Providers reported sociodemographic characteristics such as age, gender, race, ethnicity, education, and income via survey and also provided information about their program including the number of years in business, number of children in care, number of hours spent cleaning each day, whether the building was rented and built before 1978, and whether any children in their care had asthma.

#### 2.2.2. Cleaning Products

Items from the Environmental Exposures in Child Care Facilities Study [26] and the Children’s Environmental Health Network’s Eco-Healthy Child Care^®^ Checklist [41] were adapted to measure product information. Participants reported the three products that they used most often to clean, sanitize, or disinfectant the indoor areas where children are cared for. They then showed research staff these three products. Research staff abstracted the following information from the product label: (i) brand name; (ii) intended use of the product (i.e., a cleaner for removing dirt from surfaces using detergents; a sanitizer for killing bacteria using chemicals; and or a disinfectant for killing bacteria and viruses using chemicals [20]); and (iii) presence of green product certifications (i.e., Greenseal, Ecologo, US EPA Safer Choice, or Design for the Environment). A photograph was taken of the products and their labels.

#### 2.2.3. Chemical Ingredients

Product brand names and the top three chemicals listed as active ingredients were abstracted from the product labels or from photos taken on site. When exact chemical ingredients were not listed on product labels, this information was obtained through the Consumer Product Information Database (CPID) [42], manufacturer websites, and/or SDSs (formerly MSDSs or Material Safety Data Sheets).

#### 2.2.4. Chemical Designation

**Figure 1:**
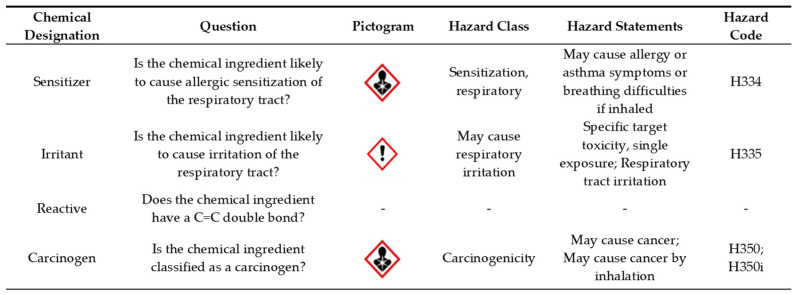
Chemical designation criteria for chemical ingredients found in cleaning products reported by family child care home (FCCH) providers. This information was adapted from the United Nation’s Global Harmonized System of Classification and Labelling of Chemicals found at: https://unece.org/transport/standards/transport/dangerous-goods/ghs-rev9-2021 (accessed on 2 March 2020).

The US Department of Labor Occupational Safety and Health Administration Hazard Communication Standard requires product labels and SDSs to classify chemicals using the Global Harmonized System (GHS) of Classification and Labeling of Chemicals [43]. The GHS classifies chemicals into hazard classes and allows for easy identification of those hazard classes using its designated pictograms. For the purpose of the current study, we were concerned with chemicals labeled with an Exclamation Mark Irritant GHS07 and Health Hazard GHS08 pictograms. Chemical ingredients that had these pictograms were further evaluated based on its GHS hazard class, statements, codes, and molecular formula structure. Molecular formulas were evaluated using the US National Library of Medicine’s ChemID*plus* [44]. A set of seven chemical designations were adapted from a nationwide survey on indoor air quality in nurseries and schools in France [6] to identify chemical ingredients that pose a potential health concern (Figure 1).

A chemical ingredient was considered a potential concern if: (i) it may cause allergic sensitization of the respiratory tract, as defined by the GHS hazard code H334; (ii) it may cause irritation to the respiratory tract, defined by the GHS hazard code H335; (iii) it has a C=C double bond that is highly reactive with ozone and may produce secondary air pollutants such as aldehydes and PM [6,22,23,39]. Sodium hypochlorite, the chemical active ingredient for bleach, and its byproducts (e.g., chloroform, chloramines, and formaldehyde) are associated with respiratory irritation and a potential trigger of asthma symptoms [8,27,28,29,30]; however, the GHS does not consider the byproducts of the chemical. Thus, we noted sodium hypochlorite during our abstraction process. In addition, given the severity of health implications, we also noted any product that was designated as a carcinogen defined by the GHS hazard codes H350 and H350i.

### 2.3. Data Analyses

Descriptive statistics were conducted for all measures, including mean and standard deviation (*SD*s) for continuous variables and frequencies and percentages for categorical or nominal variables. All analyses were performed in Microsoft^®^ Excel^®^ for Microsoft 365 MSO (Version 2112, Microsoft, Redmond, WA, USA).

## 3. Results

### 3.1. Participant Characteristics

FCCH provider and program characteristics are presented in Table 1. The average age of providers was 44 years (*SD* = 13). All participating FCCH providers were female. Nearly all were non-Hispanic (95.7%) and most identified as white (60.5%), with the majority having some college or vocational training (63.3%). Approximately one third (31.3%) of providers had a degree in early childhood education or development. The average number of years programs were in business was 11 years (*SD* = 10). FCCH programs cared for an average of seven (*SD* = 4) children ranging from birth to five years of age. The average number of self-reported daily hours providers spent cleaning their FCCH was 3 (*SD* = 3). Over a third (36.7%) of providers reported that children in their care had asthma. An additional 34.7% did not know whether children in their care had asthma.

### 3.2. Cleaning Products

Of the 132 cleaning products reported by FCCH providers, 77 distinct products were identified. The top three products reported were (i) Clorox Bleach 1, Concentrated, Regular (43.8%); (ii) Young Living Thieves Essential Oil Infused (12.5%); and (iii) Clorox Performance Bleach 2 with Cloromax (10.4%). Among the 77 distinct cleaning products, 68.8% were intended to be used as cleaners; 24.7% were intended to be used as sanitizers; and 48.1% were intended to be used as disinfectants. As noted previously, sanitizers and disinfectants contain pesticides, which may be more harmful than the detergents found in cleaners. None of the cleaning products reported had a green product certification. The 20 most commonly reported cleaning products and their intended use are presented in Table 2. The other reported products can be found in the Appendix A Table A1.

### 3.3. Chemical Usage at the Provider-Level

Of the 386 chemicals abstracted, 100 distinct or unduplicated chemicals were identified. Overall, 4.2% of providers used a product with a known sensitizer that may cause allergy or asthma symptoms or breathing difficulties if inhaled. Over a third (35.4%) of providers used a product with a known irritant that may cause irritation to the respiratory tract. Most (62.5%) providers reported using a product with a chemical that had a C=C double bond in its molecular structure that may make it highly reactive with other substances in the air to produce secondary air pollutants. Overall, most (83.3%) FCCH providers reported using a sodium hypochlorite containing product. Almost a quarter (22.9%) of providers reported using a product that contained a carcinogen. Chemical designation and class of chemical ingredients found in reported products are presented in Table 3.

## 4. Discussion

Understanding the cleaning products used in ECEs and the potential health impacts of the chemicals found in these products is critical for assessing risk and developing policies and interventions to protect children’s health. Our study provides insight into the potential health impacts of the chemicals found in cleaners used by Oklahoma FCCHs and indicates a need for improvement. FCCH providers commonly reported using chemical cleaners that contain US EPA-listed chemicals of concern for environmental exposure and asthma control, particularly products containing irritants and reactive chemicals. We found that 13% of the chemical ingredients abstracted from the cleaning products used by FCCH providers were respiratory irritants. This is lower than the previously mentioned study of 310 nurseries and schools in France that found that 49% of chemicals identified in cleaning products used were irritants. [6]. In contrast, we found much higher reactive chemical ingredients than this French study (15.0% versus 7.2%, respectively) [6]. While these differences may be due to the lack of uniformity in safety regulations or product preferences across countries, both studies found substantial use of products with irritants and reactive chemical ingredients. Similar to a study of ECEs in Washington, D.C. [5], most FCCH providers in our study (83.3%) reported using bleach or sodium hypochlorite-containing products to clean, sanitize, and disinfect. In Oklahoma, ECEs are required under the Oklahoma Administrative Code 340:110-3-304 to use a household bleach solution or a product registered with the US EPA to sanitize and disinfect. Our recent statewide survey of Oklahoma ECEs, found similar rates to the current study: 86% of centers and 92% of FCCHs reported regularly using bleach [7]. While bleach-containing-products are popular and may be effective sanitizers and disinfectants, they pose a potential respiratory concern. The byproducts (i.e., chloroform, chloramines, and formaldehyde) of bleach’s active ingredient sodium hypochlorite are associated with respiratory irritation and may trigger asthma symptoms [8,27,28,29,30]. Alternative products that have lower respiratory risk than bleach or quaternary ammonias, such as fragrance-free, non-chlorine, hydrogen peroxide-containing products [29], may result in overall healthier ECE environments for children. Hydrogen peroxide, at the concentrations found in cleaning products, presents a low risk of toxicity to humans [29] and based on a review of disinfectant efficacy covers at least 70% of ECE-relevant organisms [29]. Ready-to-use peroxide products are preferred over bleach for cleaning because they do not need to be diluted daily, have not been associated with asthma, do not cause nasal irritation, and are US EPA-registered disinfectants with the shortest dwell time, thus resulting in reduced exposure [8].

None of the providers in our study used products with a third party or green product certification, an indication that the product’s chemical ingredients are safer or less toxic. Some green product certifications, such as the US EPA Safer Choice label, require that all of the product’s chemical ingredients meet the US EPA’s safety criteria for human health and the environment including carcinogenicity, reproductive/development toxicity, and persistence in the environment without compromising specific performance standards [45]. Thus, cleaning products with a Safer Choice label are safer for: (i) individuals, families, and pets; (ii) worker’s health; and (iii) fish and the environment [45]. Educating providers about third-party certifications may influence their product purchases and reduce exposure. The recent Lifting Up Communities with Interventions and Research Study [46] conducted with women at home, found that switching to safer cleaning products decreased objectively-measured indoor air concentrations of multiple VOCs including chloroform and benzene. The promotion and provision of safer products may reduce exposures to asthma triggers and increase asthma control and warrant further investigation as an intervention for ECEs. Educating providers about green product certification may help to improve purchasing and product use behaviors.

While not the major focus of this paper, our findings regarding carcinogens is important. Almost a quarter (22.9%) of providers reported using one or more products that contained a carcinogen. Furthermore, 8.3% of reported cleaning products in our study contained a carcinogen. This is slightly higher than the previously mentioned French study, which found that 6.8% of products used contained at least one chemical identified as carcinogenic to humans by the International Agency for Research on Cancer (IARC) [6]. The differences in our findings may be due to differing standards set by the governing bodies responsible for evaluating the chemical contents of cleaning products. To illustrate, ethanol, a common chemical found in both studies, was identified as a carcinogen by the IARC, while the GHS classified ethanol as a highly flammable eye irritant. While the origins of pediatric cancer are only partly known, children have increased susceptibility to environmental carcinogens due to their unique patterns of exposure, rapid growth and development, and life expectancy [47]. Interventions to reduce children’s exposures to carcinogens in ECEs are needed.

To our knowledge, this is the first in the US to identify and characterize the chemicals in cleaning products used in FCCHs, a unique childcare setting. Nonetheless, there are limitations to note. We only studied a portion (i.e., the top three) cleaning products used to clean, sanitize, or disinfect indoor areas used for childcare. Thus, we have a limited perspective about the range of products used and may have likely underestimated the chemical burden faced by children in these facilities. Similarly, the chemical ingredients abstracted from product labels and SDSs only included the top three active chemical ingredients, excluding inactive ingredients and chemicals such as fragrances, which are complex mixtures of chemicals that are proprietary in nature. Therefore, our assessment of chemical ingredients is not comprehensive and may underestimate potential health concerns. While we observed the reported products and their labels, we did not objectively measure indoor toxicants and as a result, cannot categorize the full range of toxicants present nor determine whether levels exceed US EPA and the World Health Organization’s indoor air quality guidelines [48]. Finally, our study findings may not be generalizable since it only included 50 FCCHs in the Oklahoma City metropolitan area. Nonetheless, findings from this study underscore the need for interventions that will reduce the presence of chemicals and promote the use of alternative products that have lower respiratory and overall health risks, such as ready-to-use hydrogen peroxide products or products with green product certifications. Educating child care providers and policymakers about the potential health concerns posed by chemical ingredients in commonly used cleaning products and identifying safer cost-effective alternative products for cleaning, sanitizing and disinfection may increase environmental literacy and lead to improved policies and behaviors, and ultimately, safer childcare environments.

## 5. Conclusions

This study identified several threats to children’s respiratory and overall health posed by chemicals contained in commonly used cleaning products in Oklahoma FCCHs. While additional study is needed to better understand the chemical burdens and exposures faced by children in ECE environments, effective interventions to safeguard children’s health in ECEs are needed now.

## Figures and Tables

**Table 1:** Participant characteristics, Happy Healthy Homes (*n* = 50), Oklahoma, 2017–2018.

**Provider Characteristics**	**Mean (*SD*)**
Age (years) ^1^	44 (12.6)
	*n* (%)
Female	50 (100)
Hispanic, Latino/a, or of Spanish origin ^2^	
Yes	2 (4.35)
No	44 (95.65)
Race ^3^	
White	26 (60.47)
Black or African American	15 (34.88)
American Indian or Alaska Native	2 (4.65)
Highest level of education ^4^	
High school graduate or GED	4 (8.16)
Some college or vocational training	31 (63.27)
4-year college graduate or higher	14 (28.57)
Degree in early childhood education or development ^5^	
Yes	15 (31.25)
No	33 (68.75)
Household income ^6^	
USD 15,000 to 34,999	9 (20.45)
USD 35,000 to 49,999	8 (18.18)
USD 50,000 to 74,999	9 (20.45)
USD 75,000 or more	18 (40.91)
**Program Characteristics**	**Mean (*SD*)**
Years FCCH in business	11 (9.6)
Number of children in care	9.5 (4.2)
Number of children in care ≤ 5 years old	6.8 (3.6)
Number of hours spend cleaning FCCH each day	3.2 (2.6)
	*n* (%)
Renting building used for FCCH	12 (24.00)
FCCH building built before 1978	18 (36.00)
Children in care have asthma ^7^	
Yes	18 (36.73)
No	14 (28.57)
Don’t know	17 (34.69)

^1^ *n* = 6 missing; ^2^ *n* = 4 missing; ^3^ *n* = 7 missing; ^4^ *n* = 1 missing; ^5^ *n* = 2 missing; ^6^ *n* = 6 missing; ^7^ *n* = 1 missing.

**Table 2:** Top cleaning products reported by family child care home providers and product’s intended use, Happy Healthy Homes, Oklahoma, 2017–2018.

Product Brand Name	Cleaner ^1^	Sanitizer ^2^	Disinfectant ^3^	Total FCCHs (*n* = 48) ^4^
Clorox Bleach 1, Concentrated, Regular		X	X	21 (43.75)
Young Living Thieves Essential Oil Infused	X			6 (12.50)
Clorox Performance Bleach 2 with Cloromax		X	X	5 (10.42)
Pine-Sol Multi-Surface Cleaner, Original	X			4 (8.33)
Clorox Disinfecting Wipes 1 (Citrus and Fresh Blend)			X	4 (8.33)
Lysol All Purpose Cleaner, Complete Clean, Lemon Breeze/Cherry Blossom and Pomegranate, Pump Spray	X			4 (8.33)
Lysol brand III Disinfectant Spray, Citrus Meadows/Crisp Linen Scent, Aerosol			X	4 (8.33)
Clorox Bleach Wipes, 35 Count Canister			X	3 (6.25)
Dawn Ultra Dishwashing Liquid, Original Scent	X			3 (6.25)
Bona Hardwood Floor Cleaner Refill Soft Package	X			2 (4.17)
Clorox Commercial Solutions Clean-Up Cleaner + Bleach, Pump Spray, Professional Use	X	X	X	2 (4.17)
Clorox Splash-Less Bleach, Concentrated, Clean Linen	X	X	X	2 (4.17)
Lysol All-Purpose Cleaner	X			2 (4.17)
Lysol Disinfecting Wipes, Lemon and Lime Blossom			X	2 (4.17)
Member’s Mark Disinfecting Wipes Fresh Scent	X	X	X	2 (4.17)
Mr. Clean Multi-Purpose Cleaner with Gain Original Fresh Scent	X			2 (4.17)
Pinalen Original Multi-Purpose Cleaner	X			2 (4.17)
Pine-Sol Multi-Surface	X		X	2 (4.17)
Clorox Clean-Up Cleaner with Bleach, Original/Fresh Scent, Pump Spray	X		X	2 (4.17)
Great Value Disinfectant Spray (citrus/fresh linen scent)			X	2 (4.17)

^1^ Cleaners defined by the US EPA remove dirt and organic matter from surfaces using detergents; ^2^ Sanitizers defined by the US EPA kill bacteria on surfaces using chemicals; ^3^ Disinfectants are defined by the US EPA kills viruses and bacteria on surfaces using chemicals; Definitions found at: https://www.epa.gov/coronavirus/whats-difference-between-products-disinfect-sanitize-and-clean-surfaces (accessed on 12 August 2021); ^4^ *n* = 2 missing.

**Table 3:** Chemical designation and class of chemical ingredients found in products reported by family child care home providers, Happy Healthy Homes, Oklahoma, 2017–2018.

Chemical Designation	CAS No.	Chemical Ingredient	Chemical Classification	Total Products (*n* = 132)	Total FCCHs (*n* = 48) ^1^
Sensitizer ^2^				*n* (%)	*n* (%)
	141-43-5	Monoethanolamine	Volatile organic compound	1 (0.76)	1 (2.08)
	7647-01-0	Hydrochloric acid	Inorganic substance	1 (0.76)	1 (2.08)
Irritant ^3^					
	000079-14-1	Hydroxy acetic acid	Organic compound	4 (3.03)	4 (8.33)
	068081-81-2	Sodium dodecylbenzene sulfonate	Hydrogen carbons	4 (3.03)	4 (8.33)
	000629-25-4	Sodium laurate	Organic compound	2 (1.52)	2 (4.17)
	007722-84-1	Hydrogen peroxide	Inorganic substance	2 (1.52)	2 (4.17)
	68037-49-0	Sodium 1-tetradecanesulfonate	Hydrocarbon	2 (1.52)	2 (4.17)
	001300-72-7	Sodium xylene sulfonate	Hydrocarbon	1 (0.76)	1 (2.08)
	100-79-8	Isopropylidene glycerol	Heterocyclic compound	1 (0.76)	1 (2.08)
	107-21-1	Ethylene glycol	Alcohols	1 (0.76)	1 (2.08)
	111-76-2	Ethylene glycol monobutyl ether	Alcohols	1 (0.76)	1 (2.08)
	1336-21-6	Ammonium hydroxide	Ammonia	1 (0.76)	1 (2.08)
	151-21-3	Sodium lauryl sulfate	Alcohols	1 (0.76)	1 (2.08)
	7647-01-0	Hydrochloric acid	Inorganic substance	1 (0.76)	1 (2.08)
	87-90-1	Trichloroisocyanuric acid	Heterocyclic compound	1 (0.76)	1 (2.08)
Reactive ^4^					
	068424-85-1	Alkyl(C12-16) dimethyl benzyl ammonium chloride	Quaternary ammonium compounds	11 (8.33)	11 (22.92)
	085409-23-0	n-Alkyl (68% C12, 32% C14) dimethyl ethyl benzyl ammonium chloride	Quaternary ammonium compounds	7 (5.30)	7 (14.58)
	068989-01-5	Benzenemethanaminium, N, N-dimethyl-N-tetradecyl-, saccharinate	Quaternary ammonium compounds	6 (4.55)	6 (12.50)
	000770-35-4	Propylene glycol phenyl ether	Alcohols	5 (3.79)	5 (10.42)
	063449-41-2	C8-18-Alky dimethyl benzyl ammonium chlorides	Quaternary ammonium compounds	5 (3.79)	5 (10.42)
	068081-81-2	Sodium dodecylbenzene sulfonate	Hydrogen carbons	4 (3.03)	4 (8.33)
	025155-30-0	Benzenesulfonic acid	Organic compound	2 (1.52)	2 (4.17)
	026062-79-3	Polyquaternium-6	Quaternary ammonium compounds	2 (1.52)	2 (4.17)
	001300-72-7	Sodium xylene sulfonate	Hydrocarbon	1 (0.76)	1 (2.08)
	001328-53-6	Phthalocyanine green	Heterocyclic compound	1 (0.76)	1 (2.08)
	026172-55-4	Methylchloroisothiazolinone	Organic compound	1 (0.76)	1 (2.08)
	027176-87-0	Dodecyl benzene sulfonic acid	Hydrocarbon	1 (0.76)	1 (2.08)
	3844-45-9	FD&C Blue	Hydrocarbon	1 (0.76)	1 (2.08)
	68391-01-5	n-Alkyl (60% C14, 30% C16, 5% C12, 5% C18) dimethyl benzyl ammonium chloride	Quaternary ammonium compounds	1 (0.76)	1 (2.08)
	89-83-8	Thymol	Phenols	1 (0.76)	1 (2.08)
Carcinogen ^5^					
	068424-85-1	Alkyl(C12-16) dimethyl benzyl ammonium chloride	Quaternary ammonium compounds	11 (8.33)	11 (22.92)

^1^ *n* = 2 missing; ^2^ Chemicals that may cause allergy or asthma symptoms or breathing difficulties if inhaled, as defined by the Global Harmonization System (GHS) hazard code H334 on the chemical’s safety data sheet (SDS); ^3^ Chemicals may cause irritation to the respiratory tract, as defined by GHS hazard code H335 on the chemical’s SDS; ^4^ Chemicals with a double bonded carbon in its molecular structure that may make it highly reactive with other substances in the air to produce secondary air pollutants; ^5^ Chemicals may cause cancer, as defined by the GHS hazard code H350 and H350i on the chemical’s SDS.

## Data Availability

The data presented in this study are available on request from the corresponding author. The data are not publicly available.

## Appendix A

**Table A1:** Other products reported by family child care home (FCCH) providers and product’s intended use, Happy Healthy Homes Baseline Survey, Oklahoma, 2017–2018.

Product Brand Name	Cleaner ^1^	Sanitizer ^2^	Disinfectant ^3^	Total FCCHs (*n* = 48) ^4^
Ajax With Bleach Cleanser, Powder	X			1 (2.08)
Best choice Glass Cleaner with Ammonia	X			1 (2.08)
Bona Tile and Floor Cleaner	X			1 (2.08)
Clorox Anywhere Hard Surface Daily Sanitizing, Pump Spray		X		1 (2.08)
Clorox Bleach Gel Cleaner + Bleach, Pump Spray	X			1 (2.08)
Clorox Bleach Splash-Less Regular	X	X	X	1 (2.08)
Clorox Bleach with Pine-Sol	X			1 (2.08)
Clorox Commercial Solutions Germicidal Bleach 4, Regular, Professional Use	X	X	X	1 (2.08)
Clorox Fraganzia Multi-Purpose Cleaner, Spring (Primavera)	X		X	1 (2.08)
Clorox Healthcare Bleach Germicidal Wipes, Professional Use	X	X	X	1 (2.08)
Clorox Splash-Less Bleach, Concentrated, Fresh Meadow		X	X	1 (2.08)
Comet with Bleach Disinfectant Cleanser	X		X	1 (2.08)
Dawn Simply Clean Dishwashing Liquid Dish Soap, Original Scent	X			1 (2.08)
Dawn Ultra Dishwashing Liquid Dish Soap, Original		X		1 (2.08)
DG Home Disinfectant Spray Fresh Linen Scent			X	1 (2.08)
Fabuloso Multi-Purpose Cleaner, Lavender	X			1 (2.08)
Fresh Scent Disinfecting Wipes	X	X	X	1 (2.08)
Freshine Disinfecting Wipes Lemon Scented			X	1 (2.08)
Good & Clean disinfecting wipes			X	1 (2.08)
Great Value Cleaning Bleach	X	X	X	1 (2.08)
Great Value Lavender Low-Splash Bleach		X	X	1 (2.08)
Home Select ultra dish soap (cherry blossom)	X			1 (2.08)
* Kustom Kleen	-	-	-	1 (2.08)
Lysol Brand Clean & Fresh Multi-Surface Cleaner, Sparkling Lemon & Sunflower	X			1 (2.08)
Lysol Brand II Disinfecting Wipes, Lemon & Lime Blossom			X	1 (2.08)
Lysol Brand II Disinfecting Wipes, Ocean Fresh			X	1 (2.08)
Lysol Brand Multi-Purpose Cleaner with Hydrogen Peroxide, Citrus Sparkle Zest, Pump Spray	X			1 (2.08)
Lysol Clean and Fresh Multi Surface cleaner (Cherry blossom & Pomegranate)	X	X	X	1 (2.08)
Lysol Max Cover Disinfectant Mist, Lavender Field, Aerosol			X	1 (2.08)
Lysol Power & Free Multi-Purpose Cleaning Wipes with Hydrogen Peroxide, Oxygen Splash	X			1 (2.08)
Lysol Power Toilet Cleaner		X	X	1 (2.08)
Lysol Professional Disinfectant Spray, Fresh, Aerosol, Professional Use			X	1 (2.08)
Melaleuca-Clear Power Glass Cleaner	X			1 (2.08)
Member’s Mark Commercial Lemon Fresh Cleaner	X		X	1 (2.08)
Member’s Mark Disinfectant Wipes Lemon Scent	X	X	X	1 (2.08)
Method Cleaning Products Squirt + Mop Wood Floor Cleaner Almond	X			1 (2.08)
Mop & Glo Multi-Surface Floor Cleaner, Fresh Citrus Scent	X			1 (2.08)
Mr. Clean Multi-Purpose Cleaner with Febreze Meadows and Rain	X			1 (2.08)
Mrs. Meyers Multi-Surface Cleaner-Geranium	X			1 (2.08)
OdoBan Eliminates Odors, Continuous Spray, Disinfectant Fabric & Air Freshener, Eucalyptus Scent-10001, Aerosol			X	1 (2.08)
Palmolive Ultra Oxy, Power Degreaser, Dishwashing Hand Liquid	X			1 (2.08)
Pine-Sol Multi-Surface Cleaner, Lemon Fresh Scent	X			1 (2.08)
Pine-Sol Multi-Surface Cleaner, Sparkling Wave Scent	X			1 (2.08)
Pledge Commercial Line, Lemon Clean, Aerosol, Professional Use	X			1 (2.08)
Purell Foodservice Surface Sanitizer, Pump Spray, Professional Use		X	X	1 (2.08)
Rejuvenate Floor Cleaner	X			1 (2.08)
Resolve Large Area Carpet Cleaner Machine Solution—2X Concentrate	X			1 (2.08)
Simple Green Stone Cleaner & Polish, Pump Spray	X			1 (2.08)
Soft Scrub Commercial Cleanser with Bleach, Professional Use	X	X	X	1 (2.08)
Sol-u-guard Botanical			X	1 (2.08)
Sol-u-mel stain remover (lemon blossom)	X			1 (2.08)
Streak Free Glass Cleaner MPC	X			1 (2.08)
Swiffer disposable mopping pad	X			1 (2.08)
Swiffer Sweeper Wet Mopping Cloths with Febreze Freshness, Lavender Vanilla & Comfort, 24 count	X			1 (2.08)
The Home Store glass cleaner	X			1 (2.08)
Thieves	X			1 (2.08)
Windex Multisurface Disinfectant Cleaner, Glade Rain shower Scent, Pump Spray			X	1 (2.08)

^1^ Cleaners defined by the US EPA remove dirt and organic matter from surfaces using detergents; ^2^ Sanitizers defined by the US EPA kill bacteria on surfaces using chemicals; ^3^ Disinfectants are defined by the US EPA kills viruses and bacteria on surfaces using chemicals; Definitions found at: https://www.epa.gov/coronavirus/whats-difference-between-products-disinfect-sanitize-and-clean-surfaces (accessed on 12 August 2021); ^4^ *n* = 2 missing; * Could not access product label information.
